# Interplay between Peripheral and Central Inflammation in Obesity-Promoted Disorders: The Impact on Synaptic Mitochondrial Functions

**DOI:** 10.3390/ijms21175964

**Published:** 2020-08-19

**Authors:** Marianna Crispino, Giovanna Trinchese, Eduardo Penna, Fabiano Cimmino, Angela Catapano, Ines Villano, Carla Perrone-Capano, Maria Pina Mollica

**Affiliations:** 1 Department of Biology, University of Naples Federico II, 80126 Naples, Italy; crispino@unina.it (M.C.); giovanna.trinchese@unina.it (G.T.); eduardo.penna@unina.it (E.P.); fabiano.cimmino@unina.it (F.C.); angelacatapano@me.com (A.C.); 2 Department of Pharmacy, University of Naples Federico II, 80131 Naples, Italy; perrone@unina.it; 3 Department of Experimental Medicine, University of Campania “Luigi Vanvitelli”, 80138 Naples, Italy; ines.villano@unicampania.it; 4 Institute of Genetics and Biophysics “Adriano Buzzati Traverso”, National Research Council (CNR), 80131 Naples, Italy

**Keywords:** high fat diet, mitochondria, synaptic plasticity, inflammation, neuroinflammation, neurological disorders, Alzheimer’s disease, Parkinson’s disease, bipolar disorders and schizophrenia

## Abstract

The metabolic dysfunctions induced by high fat diet (HFD) consumption are not limited to organs involved in energy metabolism but cause also a chronic low-grade systemic inflammation that affects the whole body including the central nervous system. The brain has been considered for a long time to be protected from systemic inflammation by the blood–brain barrier, but more recent data indicated an association between obesity and neurodegeneration. Moreover, obesity-related consequences, such as insulin and leptin resistance, mitochondrial dysfunction and reactive oxygen species (ROS) production, may anticipate and accelerate the physiological aging processes characterized by systemic inflammation and higher susceptibility to neurological disorders. Here, we discussed the link between obesity-related metabolic dysfunctions and neuroinflammation, with particular attention to molecules regulating the interplay between energetic impairment and altered synaptic plasticity, for instance AMP-activated protein kinase (AMPK) and Brain-derived neurotrophic factor (BDNF). The effects of HFD-induced neuroinflammation on neuronal plasticity may be mediated by altered brain mitochondrial functions. Since mitochondria play a key role in synaptic areas, providing energy to support synaptic plasticity and controlling ROS production, the negative effects of HFD may be more pronounced in synapses. In conclusion, it will be emphasized how HFD-induced metabolic alterations, systemic inflammation, oxidative stress, neuroinflammation and impaired brain plasticity are tightly interconnected processes, implicated in the pathogenesis of neurological diseases.

## 1. Overnutrition and Altered Energy Homeostasis

Genetic predisposition, sedentary lifestyle, eating habits, environmental factors and altered metabolism are the main and convergent contributors in the onset of obesity, a well-known complex and multifactorial pathology that has reached pandemic proportions. Obesity incidence has increased very quickly over the past 30 years, suggesting that both behavioural and environmental factors, in particular greater availability and consumption of food with high fat content, have strongly fuelled this condition [1]. High fat diet (HFD)-induced obesity is related to several pathological conditions such as diabetes, cardiovascular diseases, hypertension, liver diseases, some forms of cancer (colon, gallbladder, breast etc.) and neurodegenerative diseases [2]. The common feature of all obesity-induced metabolic diseases is chronic low-grade inflammation that is tightly related to the physiopathology of adipose tissue [3].

**Figure 1:**
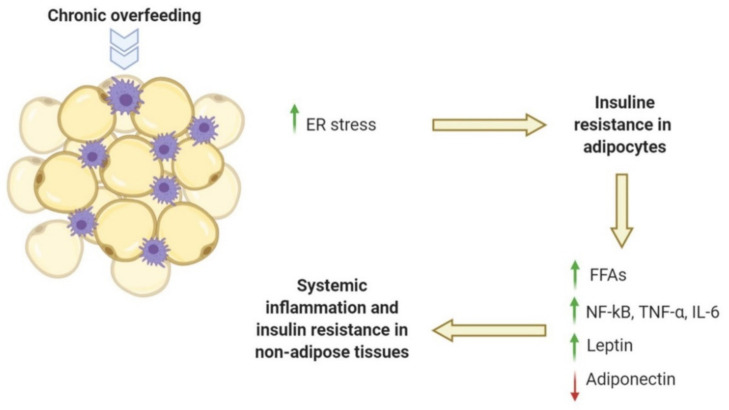
Chronic overfeeding causes an increase in adipose tissue depots leading to systemic inflammation. Adipocyte hypertrophy is a possible stress condition for the endoplasmic reticulum (ER), which in turn activates inflammatory pathways and causes insulin resistance in adipose tissue. Insulin-resistant adipocytes induce an increase in circulating free fatty acids (FFAs), synthesis of pro-inflammatory cytokines, and an alteration of leptin and adiponectin levels, triggering ectopic accumulation of lipids, systemic inflammation and insulin resistance in non-adipose tissues. The red arrow indicates decrease, and the green arrows indicate increase.

Adipocytes control energy homeostasis, modulating energy storage and utilization; sense energy needs; and secrete hormones and adipokines that exert a large array of biological effects on metabolism, homeostasis and inflammation. Therefore, the classical view of adipose tissue as an inert fat storage tissue has been rapidly overcome and it is currently considered obsolete [4,5,6]. Chronic overfeeding causes an increase in adipose tissue depots related to both hyperplasia and hypertrophy of fat cells. When the adipose tissue expandability is low, the adipose depots are characterized by an increased presence of hypertrophic adipocytes. Adipocyte hypertrophy is a possible stress condition for the endoplasmic reticulum (ER), which in turn activates inflammatory and apoptotic pathways and causes insulin resistance (IR) in adipose tissue [3,7]. Insulin-resistant adipocytes, being more lipolytic and less liposynthetic, induce an increase in circulating free fatty acids (FFAs). These FFAs may also activate Toll-like receptors 4 (TLR4), that induce Nuclear Factor kappa-light-chain-enhancer of activated B cells (NF-κB) translocation to the nucleus and subsequent synthesis of pro-inflammatory cytokines as tumour necrosis factor-α (TNFα) and interleukin-6 (IL-6) [8,9,10]. This complex signalling amplifies IR, lipolysis and inflammation in whole adipose tissue concomitantly with altered circulating leptin and adiponectin levels. Leptin and adiponectin, two adipose tissue-derived hormones, are involved not only in glucose and lipid metabolism, controlling energy homeostasis, but also in the modulation of inflammation [11,12]. With the accumulation of fat mass, leptin levels increase while adiponectin decreases. In some animal models, the increased adiponectin levels were found to correlate with insulin sensitivity and lower risk of metabolic syndrome [4,5]. The altered circulating leptin and adiponectin levels lead to leptin resistance and to a decreased lipid oxidation in non-adipose tissues, thereby triggering ectopic accumulation of lipids, lipotoxicity and IR [3]. In particular, the ratio adiponectin/leptin negatively correlates with systemic inflammation and IR more closely than adiponectin or leptin alone and represents a hallmark of adipose tissue dysfunction [13] (Figure 1).

The pleiotropic inflammatory-regulator role exerted by these adipokines was also attributed to their ability to activate AMP-activated protein kinase (AMPK), a sensor of the cellular energy status able to modulate multiple metabolic pathways [14,15]. Leptin and adiponectin, through AMPK activation, also stimulate glucose utilization and fatty acid (FA) oxidation [16]. Indeed, AMPK controls cellular energy homeostasis by turning on catabolic pathways (glycolysis, fatty acid oxidation and mitochondrial biogenesis) and by inhibiting anabolic pathways (gluconeogenesis, glycogen, FA and protein synthesis) [17]. AMPK modulates cell metabolism based on the availability of nutrients and their capacity to produce ATP through mitochondrial oxidative phosphorylation. Chronic low-grade inflammation induced by obesity reduces AMPK activity in multiple tissues including skeletal muscle [18,19,20], liver [21,22,23] and adipose tissue [23,24], with a mechanism still partly unknown but probably involving altered mitochondrial fatty acid oxidation.

## 2. Mitochondria Dysfunctions, Oxidative Stress and Inflammation

Mitochondria are defined as “powerhouses” since they supply the cell with ATP produced by oxidative phosphorylation, a process involving the flow of electrons through the electron transport chain from high-energy substrates to oxygen. They are essential organelles in the cell not only for their bioenergetic role but also because they are involved in ion homeostasis, in several metabolic pathways, in apoptosis and programmed cell death, in the synthesis of key molecules related to inflammation, and in reactive oxygen species (ROS) production and consumption. Therefore, it is no surprise that mitochondrial dysfunctions are associated with inflammation and other energy-dependent disturbances, where the cellular oxidative damages are caused by generation of ROS exceeding the natural antioxidant activity [25]. Nonetheless, it is important to underline that mitochondrial dysfunction can be not only the cause but also the consequence of inflammatory processes and elicits metabolic adaptations that might be protective or might become progressively detrimental [26]. Thus, altered mitochondrial functions are part of the wide spectrum of metabolic changes induced by overnutrition-dependent low-grade inflammation [18,21]. In particular, mitochondrial dysfunction in liver and skeletal muscle leads to reduced FAs oxidation, impaired glucose homeostasis, increased ectopic lipid accumulation and decreased insulin sensitivity [15,19]. This is a hallmark of IR and type 2 diabetes [27,28,29,30,31]. The mitochondria of people with obesity are different from those of lean individuals, since they display altered morphology and bioenergetics, increased lipid peroxidation, decreased ATP content and reduced fatty acid oxidation. Mitochondrial β-oxidation is primarily responsible for the degradation of long, medium and short chain FAs [32,33]. Excessive fat consumption triggers β-oxidation of FFAs at the mitochondrial level, leading to an excessive flow of electrons using cytochrome c oxidase, which increases the accumulation of ROS. These excessive ROS induce inflammatory response and activate the transcription factor NF-κB. In turn, NF-κB stimulates the production of a series of pro-inflammatory molecules such as interferon-γ, TNF-α and inducible nitric oxide synthase [34]. These differences in mitochondrial functions among obese and lean subjects might promote development and progression of obesity.

Several evidences indicate that various aspects of mitochondrial biology and homeostasis, such as mitochondrial biogenesis, regulation of the mitochondrial network and mitochondrial quality control, are specifically regulated by AMPK [35]. AMPK also controls mitochondrial FA oxidation, stimulating lipid β-oxidation and inhibiting the first step in FAs synthesis. The activation of AMPK decreases the expression of lipogenic genes and increases the phosphorylation of acetyl-CoA carboxylase (ACC), resulting in its inactivation. ACC, an enzyme involved in the initial phase of FA synthesis, leads to a reduction in malonyl-CoA when it is inactivated. Malonyl-CoA regulates FA oxidation through the inhibition of Carnitine palmitoyl-transferase (CPT). Thus, the inhibition of malonyl-CoA results in activation of CPT activity. CPT when activated increases entry of long-chain FAs into the mitochondria [36].

**Figure 2:**
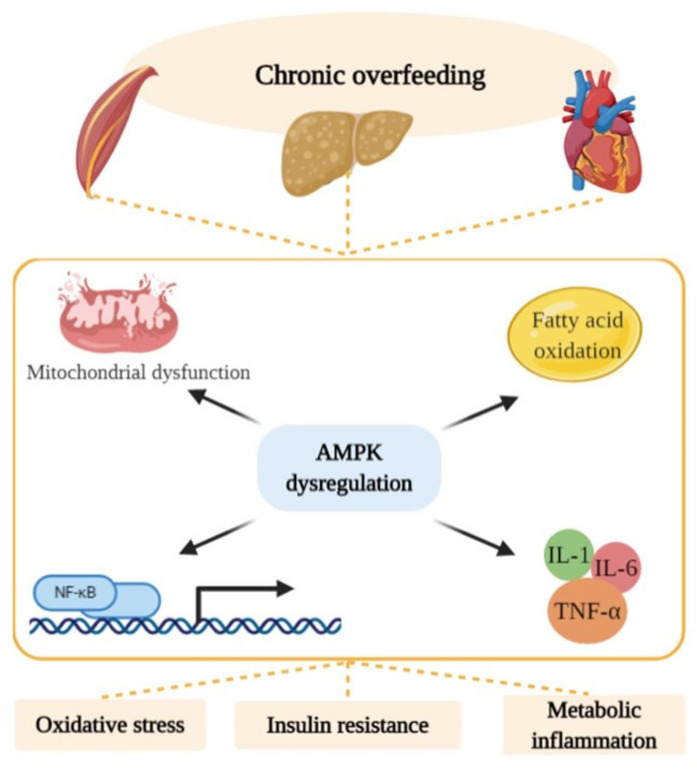
Chronic overnutrition and ectopic lipid accumulation reduces AMP-activated protein kinase (AMPK) activity in multiple non-adipose tissues. The prolonged inactivation of AMPK may be responsible, at least in part, for alterations in mitochondrial function, fatty acid (FA) oxidation, activation of nuclear factor kappa-light-chain-enhancer of activated B cells (NF-κB) signalling and the consequent appearance of a low-grade metabolic inflammation, oxidative stress and insulin resistance.

AMPK is also a potent counter-regulator of inflammatory pathways [14,15], and its actions are independent of its effects on glucose and lipid metabolism [14]. Activation of AMPK, through its signalling network, can suppress the NF-κB pathway, inhibiting the synthesis of proinflammatory cytokines and promoting the expression of interleukin-10 (IL-10) in macrophages [37]. Adiponectin and leptin levels may be also regulated by AMPK participating in anti-inflammatory response [38]. Through this complex signalling pathway, AMPK ameliorates the proinflammatory phenotype associated with dysmetabolic and aging-related diseases. Interestingly, in adipose tissue, skeletal muscle and liver of obese subjects, the AMPK activity was found to be decreased [39]. This decreased activity may be responsible for the activation of NF-κB signalling and the consequent appearance of a low-grade, metabolic inflammation [37]. The prolonged inactivation of AMPK may be responsible, at least in part, for alterations in FA oxidation, leading to lipid overloaded hypertrophic adipocytes associated with inflammation and IR [40]. Thus, AMPK can be hypothesized as a potential target for treating metabolic dysfunctions, including diabetes, obesity and fatty liver diseases, and cancer, which is often associated with changes in metabolism. Indeed, several results show that the activation of AMPK, by appropriate diet supplementation, reduces obesity and IR [18,19,41] (Figure 2).

## 3. Interplay between Obesity and Aging: Inflammation and Neuroinflammation

**Figure 3:**
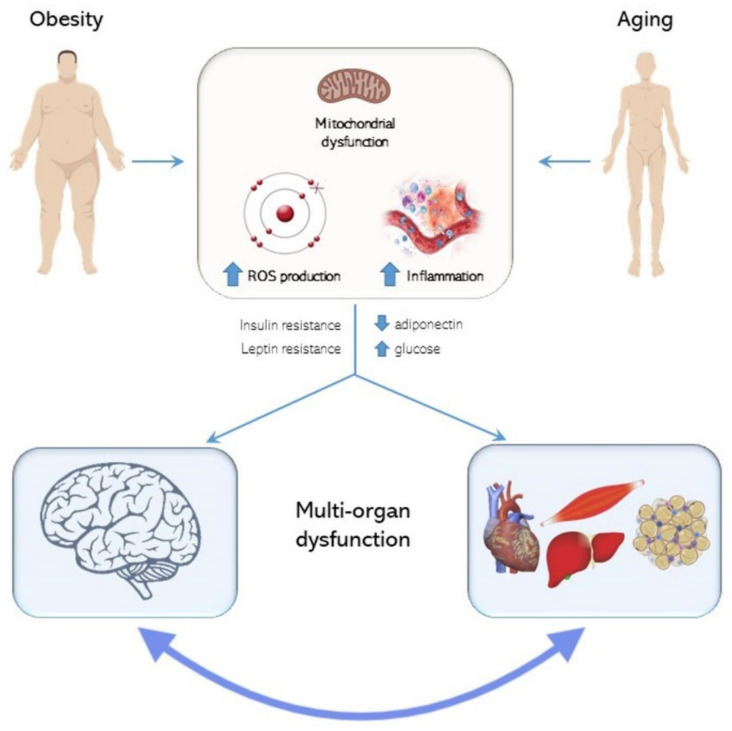
Chronic overnutrition and aging negatively affect peripheral organs and the central nervous system by mitochondria dysfunction, increased oxidative stress and inflammation. The metabolic alterations associated with obesity include insulin and leptin resistance, decreased adiponectin and increased glucose levels.

HFD-induced obesity is characterized by low-grade inflammation, IR, oxidative stress and mitochondrial dysfunctions that are also typical features of aging. The normal aging process includes impairment of energy homeostasis, leading to progressive increase in adiposity [42]. Therefore, overweight is frequently observed in middle-aged subjects (age-related obesity), and it may accelerate the aging processes [43]. In general, obesity and aging share several pathophysiological features characterized by a progressive organ dysfunction that alters the maintenance of homeostasis [44,45]. Aged rats were reported to develop leptin resistance that is typically associated with obesity [46]. In general, multi-organ damage due to inflammation and oxidative stress, hallmarks of both aging and obesity, highlight that these two processes are tightly interconnected [47]. The systemic impact of metabolic inflammation involves also the central nervous system [48]. The brain has been considered for a long time to be an organ protected from systemic inflammation by the blood–brain barrier, but more recent data indicate a tight association between obesity and neurodegeneration. Indeed, obesity-related pathological consequences, such as insulin and leptin resistance, mitochondrial dysfunction and ROS production, may anticipate and accelerate the physiological aging processes that involve also higher susceptibility to neurodegenerative diseases. In particular, it has been demonstrated that the age at which HFD feeding starts and the diet duration are both important factors in determining development of obesity, underlining that the interaction between age and HFD potentiates their negative effects on metabolic parameters [49]. Since obese people, due to neuroinflammation, have increased risk of developing brain disease, molecules acting as regulators in the cross talk between adipose tissue and the central nervous system (CNS) might represent key elements to help cure these diseases [50] (Figure 3).

## 4. Overnutrition-Dependent Neuroinflammation and Hypothalamic AMPK Activation

A brain region well-known to be affected by food habits is the hypothalamus. In particular, it has been demonstrated that both the consumption of HFD and the aging process evoke an inflammatory response in the hypothalamus, inducing resistance to insulin and leptin. Therefore, hypothalamic inflammation has been identified as a crucial step not only in the development of obesity with a defective control of food intake and energy expenditure but also in the aging processes. The hypothalamic inflammatory responses to dietary fat and consequent metabolic stress have been observed both in mice and humans. Several evidences suggested that these responses are mediated by TLR signalling, which results in the activation of NF-κB and production of inflammatory cytokines, such as IL-1β, IL-6, and TNF-α [51,52,53]. Therefore, the dysfunctions of hypothalamic signalling, involving especially IKKβ/NF-κB, is a general cause of multiple neural diseases and contributes to the pathological features related to overnutrition [54]. The hypothalamic IKKβ/NF-κB upregulation, induced by HFD, is also associated with diminished hypothalamic insulin and leptin signal transduction, contributing to defective food intake [55,56]. Interestingly, in rodent models, it was reported that inflammation in hypothalamus is an early event, within 1 to 3 days of HFD exposure, prior to substantial weight gain, while in peripheral tissues, it develops over weeks to months of HFD feeding [57]. These HFD-induced early hypothalamic inflammation and oxidative stress were initially counteracted by the antioxidant defences that balance the ROS levels [49].

AMPK plays a major role in hypothalamic mechanisms for controlling energy homoeostasis and food intake [58]. This role is the result of the integration of orexinergic and anorexinergic molecules, such as leptin, adiponectin, ghrelin and insulin, with hypothalamic neuronal networks [59]. In physiological conditions, AMPK activity is inhibited in various hypothalamic regions by the anorexigenic hormone leptin and by insulin. It is noteworthy that the effects of leptin are tissue-specific, resulting in reduction of the appetite centrally, mediated by inactivation of AMPK, and in the increase of the peripheral FAs consumption mediated by stimulation of AMPK activity. Unlike leptin, adiponectin stimulates AMPK activity both in CNS and peripheral tissues. In liver and skeletal muscle, AMPK activation by adiponectin leads to stimulation of glucose uptake and fatty acid oxidation, modulating mitochondrial functions [11]. In the CNS, adiponectin receptors have been identified in several regions including the hypothalamic arcuate and lateral nuclei [60], suggesting a role played by this adipokine in feeding and energy expenditure. Although physiological activation of AMPK in peripheral tissues promotes FA oxidation and insulin sensitivity, in the pathological inflammatory state, chronic activation of AMPK activity in the hypothalamus causes obesity by inducing hyperphagia in both humans and rodents [58]. It was recently demonstrated that long-term administration of HFD induces persistent activation of AMPK in the hypothalamus that is still active after 12 and 18 weeks of treatment. Interestingly, it was reported that hypothalamic inflammation occurs within few days of HFD treatment, suggesting that the negative effects of HFD start in the hypothalamus and affect peripheral tissues only after prolonged consumption of fat [49].

## 5. Overnutrition and Synaptic Plasticity

Plasticity of the brain is the ability of neurons to modulate themselves in response to stimuli for storage of new information [61]. To adapt to the changing environment, the neurons undergo a molecular and structural rearrangement of subcellular compartments such as dendrites, axon and nerve endings depending on the modulation of several biochemical pathways [62,63]. It is well established that synaptic plasticity occurs through activity-dependent modification of the number and/or strength of synaptic connections [64]. The plasticity of neuronal circuits occurs not only during embryonic development but also throughout childhood and adulthood. It was reported that consumption of HFD, even for a short period of time, can negatively affect cognitive functions [65] and that adolescence periods represent a window of higher sensitivity to the effects of HFD [66]. Indeed, it has been demonstrated that, during this period, the consumption of HFD is linked to change in synaptic plasticity, leading to impairment of cognitive functions [67].

Brain plasticity is strictly influenced not only by mRNA encoding for proteins but also by the information contained in the noncoding RNA (ncRNA). Indeed, it is now accepted that the majority of the mammalian genomes are transcribed into ncRNA [68]. These RNAs, which mostly consist of long ncRNAs (lncRNAs) and small ncRNAs, are abundantly expressed in the brain, where they play a role in synaptic plasticity during development [69,70] as well as in memory formation [71,72]. In a recent study, using RNA sequencing of coding and noncoding RNAs, it was demonstrated that the expression levels of mRNAs related to neurogenesis, synaptic plasticity and calcium signalling were decreased after HFD. Interesting, several ncRNAs were also differentially expressed in HFD mice compared to control [73], suggesting a crucial role played by ncRNAs in linking nutritional influence, metabolic disease and neuronal plasticity.

Several studies indicate that HFD may activate signalling pathways with deleterious effects in various brain regions [74]. In particular, in the hypothalamus, consistently with early inflammation onset and activation of AMPK, HFD has a rapid and profound effect on neuronal plasticity. This area of the brain undergoes rapid structural changes in response to HFD within 3 days of administration, showing alterations of many cytoskeletal proteins involved in neuronal remodelling and synaptic plasticity [75]. A strong loss of synapses was observed on pro-opiomelanocortin (POMC) neurons of hypothalamic arcuate nucleus after HFD, associated with increased glial ensheathment of the POMC perikarya [76].

Consumption of HFD can critically affect synaptic functions also in the prefrontal cortex (PFC), a brain area playing a key role as food behaviour regulator [67,77]. Alteration of PFC may induce eating disorders and may contribute to the development of obesity [77]. The role of PFC in controlling food behaviour is exerted, at least in part, through the modulation of γ-aminobutyric acid (GABA) neurotransmission [78]. GABA, using especially GABAB receptors, is involved in the cognitive choice of selecting the type, quantity and quality of food [79,80,81]. Some studies demonstrated a decrease of GABA levels in PFC after HFD exposure [78,82], suggesting a role of this neurotransmitter in eating disorders. Moreover, dysregulation of micro RNAs (miRNAs) expression was observed in PFC and the targets of these altered miRNAs included the transcripts for essential neural functions such as axon guidance [67].

The deleterious effects of HFD have also a profound impact on the hippocampus, a brain region playing a crucial role in learning and memory [83,84]. Hippocampal inflammation was reported in adolescent rats in response to HFD [85], with partial inhibition of long-term potentiation (LTP) observed in hippocampal slices after 48 hrs of HFD treatment [86]. In addition, HFD is able to trigger the release of exosomes from microglia that, in turn, may mediate alteration of dendritic spines [87]. Interestingly, HFD also induces a decrease in PSD-95 protein levels and dendritic spine density in mice hippocampus, with an increase in pAMPK levels [88]. Synaptic activation in response to different stimuli leads to the expression of immediate early genes (IEGs) that are responsible for long-term memory formation. The expression of IEGs requires the activation of AMPK, that also influences the neuronal energetic status. Therefore, AMPK may represent a link between memory process and metabolic control [89]. In addition, increase in AMPK activation with age is a key factor leading to age-related decline of hippocampal neurogenesis and, thus, increased susceptibility to age-associated neurological diseases [90]. Thus, it is possible to hypothesize that HFD-induced increased activation of AMPK in the hippocampus exacerbates age-related decline, contributing to the synaptic dysfunctions observed in neurodegenerative diseases.

### 5.1. Overnutrition and BDNF

BDNF (Brain-derived neurotrophic factor) has also been proposed as a regulator of energy balance and synaptic plasticity. This neurotrophin plays a key role in the physiology and pathology of the brain [91], and it is involved in different aspects of neuronal plasticity, such as synaptogenesis, dendritic growth and branching, and modulation of excitatory and inhibitory neurotransmission [92,93]. The BDNF modulation of neuronal plasticity is mediated by the activation of synapsin I, a synaptic vesicle protein involved in exocytosis of neurotransmitters and maintenance of the synaptic contacts [94,95]. BDNF plays also an important role in energy metabolism, regulating food intake and weight gain and increasing locomotor activity [96]. Interestingly, in adult HFD-treated mice, impaired hippocampal synaptic plasticity and reduced cognitive abilities are both linked to brain BDNF levels [97,98].

HFD-dependent neuroinflammation and oxidative stress play a key role in the alteration of the BDNF levels not only in the hippocampus but also in several brain regions [48,99,100,101]. In particular, BDNF may be linked to neuroinflammation through NF-κB, although the exact regulatory mechanisms are not completely understood [91,102].

BDNF has a short-term effect in synaptic plasticity, influencing post-translation modification of proteins already available at the synapse, and long-term effects including modulation of the synaptic system of protein synthesis [103]. Thus, it is possible to hypothesize that the decreased BDNF expression in synaptic regions of HFD-treated mice [48] is linked to a diminished synaptic protein synthesis associated to altered mitochondrial activity. It is noteworthy that this local system of protein synthesis plays a key role in neuronal plasticity, responding with rapid and subtle modulation of the proteome to remodel the synaptic regions in response to stimuli [104,105,106,107,108,109,110]. Interestingly, mitochondria activity has been demonstrated to be directly linked to the synaptic system of protein synthesis [111,112].

### 5.2. Overnutrition and Brain Mitochondrial Dysfunctions

**Figure 4:**
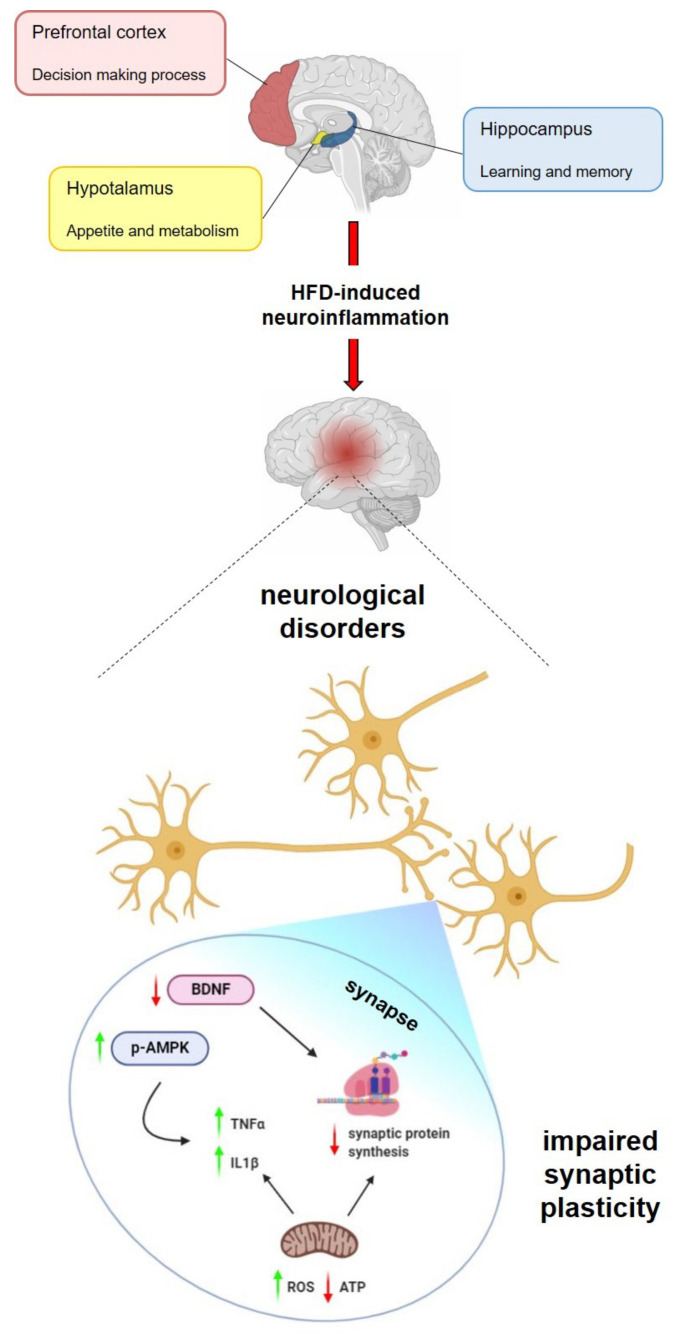
Overnutrition-induced neuroinflammation: molecular mechanisms. The overnutrition-induced neuroinflammation directly affects the hypothalamus (controlling appetite and metabolism), hippocampus (involved in learning and memory) and prefrontal cortex (playing a crucial role in decision making processes) and contributes to neurological disorders. The negative effects of high fat diet (HFD) is more pronounced in the synaptic region of the neuron leading to impaired synaptic plasticity. AMPK and Brain-derived neurotrophic factor (BDNF) mediate the interplay between energetic impairment and synaptic integrity. In the synapse, BDNF and altered mitochondrial functions may contribute to a diminished local protein synthesis. The red arrows indicate decrease, and the green arrows indicate increase.

The brain has the highest energy requirements compared to any other organ within the body. Indeed, although it is only 2% of total body weight, it receives 15% of cardiovascular outputs and consumes nearly 20% of total body oxygen and 25% of total body glucose. This great amount of energy, necessary for neuronal populations to transmit nervous signals and to conduct extensive antero- and retrograde transport along axons, is provided by functions of the mitochondria [113]. Neuronal mitochondria are differently distributed in various brain regions, and within the neuron, they have discontinuous distribution along dendrites, dendritic spines, axon and presynaptic terminals [114]. Specifically, mitochondria located in synapses play a critical role in sustaining synaptic functions, providing energy for numerous processes such as exocytosis, neurotransmitter reuptake, receptor and ion channel functioning [115,116], as well as in fuelling local protein synthesis necessary for synaptic plasticity [117]. Accordingly, many studies have shown the existence of a crucial link between dysfunction of synaptic mitochondria (that do not satisfy the synaptic high energy request) and oxidative stress, neuroinflammation and alteration of synaptic plasticity with consequent synaptic failure. These complex alteration patterns may represent the bases of many neurodegenerative diseases and others forms of cognitive impairment that imply synaptic dysfunctions [118,119,120,121,122,123]. Accordingly, it was recently reported that the HFD-dependent mitochondrial dysfunction, neuroinflammation and oxidative stress are particularly pronounced in the synaptic regions of mice brain cortex [48] (Figure 4).

#### 5.2.1. Alzheimer’s Disease

It is noteworthy that mitochondrial dysfunction and neuroinflammation are implicated in the neuronal loss that characterizes several neurodegenerative diseases including dementia and Alzheimer’s disease [124]. HFD has been proposed as a risk factor for AD [125]. Indeed, it strictly modulates amyloid precursor protein (APP) and tau protein, the two hallmarks of AD [126,127]. In particular, long-term treatment with HFD in young mice results in increased APP levels in hippocampus and adipose tissue, concomitant with increased inflammatory status. Interestingly, APP itself may contribute to neuroinflammation [128]. HFD modulates not only APP but also amyloid-β peptide levels [129]. Indeed, in the hippocampus of young mice, even a short period of consumption of an HFD is associated with increased β-amyloid; phosphorylated Tau; increased levels of proinflammatory cytokines, such TNFα and IL1β; and activation of AD-related genes [130]. Phosphorylation of both AMPK and mammalian target of rapamycin (mTOR) was observed in AD brains, together with hyperphosphorylation of tau. It was hypothesized that the strong activation of these two metabolic axes in AD brains is concomitant with the increase of oxidative stress [131]. Several lines of evidence suggest that mitochondrial alterations are another key factor in the synaptic failure characterizing the disease. Misfolded β-amyloid and tau in AD destabilized the outer mitochondrial membrane through direct interaction, causing an increase in ROS production and release of proapoptotic cytokines [132,133]. Aggregation of β-amyloid in neurons inhibits key enzymes in the mitochondrial metabolic chain, which leads to damage in electron transport chain, ATP production and mitochondrial membrane potential [134]. In conclusion, the deregulation of APP and Tau protein observed in HFD may affect mitochondrial functions, altering in turn inflammatory state and energy availability.

Microtubule-associated proteins, including Tau, are involved in the transport of mitochondria from the cell body to the synaptic area through the axon. Hyperphosphorylation of Tau, occurring in AD, alters mitochondria localization, leading to axonal dysfunction and loss in mitochondrial translocation to the synapse, causing synaptic energy deficiency [135,136]. The altered energy demand at the synapses can affect the synaptic proteins synthesis, which was demonstrated to be deregulated in the brain cortex of AD animal model [137].

#### 5.2.2. Parkinson’s Disease

Parkinson’s Disease (PD), the second most common neurodegenerative disorder after AD, is characterized by tremors, bradykinesia, rigid muscles, and impaired posture and balance. It mostly affects old people with an incidence of 1–3% in individuals over 65 that rises to 4–5% in people over 85 [138]. PD is characterized by loss of dopaminergic neurons in the substantia nigra pars compacta and by the intraneuronal presence of Lewy bodies containing aggregates of alpha-synuclein, neurofilaments and ubiquitin [139]. The cause of neurodegeneration in PD is not completely understood, but it has been repeatedly demonstrated the involvement of mitochondrial dysfunction in the pathway leading to the selective neuronal loss associated with the disease. Mitochondrial dysfunction implies respiratory chain impairment, that is one of the key features of PD. In addition, alteration of the mitochondria ability to clear oxidized proteins also contributes to PD neurodegeneration [140]. Interestingly, several PD-related genes encode proteins that are relevant for mitochondrial homeostasis, for instance, alpha-synuclein, the E3 ubiquitin ligase Parkin and PTEN-induced putative kinase 1 (PINK1; [141]). In particular, PINK1/Parkin-mediated mitophagy is one of the mechanisms involved in quality control of mitochondria, with PINK1 sensing mitochondrial depolarization, ROS and protein misfolding and triggering mitophagy [142]. The interruption of this PINK1/Parkin pathway may affect the ability of mitochondria to clear oxidized proteins, potentially contributing to the mitochondrial dysfunction observed in PD [143]. One of the early events characterizing PD is the abnormal alpha-synuclein deposition. The mechanism by which accumulation of this protein contributes to the death of dopaminergic neurons in PD is currently unclear, but there is growing evidence that mitochondrial dysfunction may play a role. Alpha-synuclein has been shown to accumulate in mitochondria, impairing various functions of the organelles [144], and several studies suggest bidirectional relationship between alpha-synuclein and the mitochondria. Disturbances in the dynamics of this interaction may result in a shift of the properties of alpha-synuclein from neuroprotective to neurotoxic [140]. It can be concluded that mitochondrial activity plays a crucial role in the development of PD, although it is still questionable whether its alteration is a cause or consequence of neuronal loss associated to PD [145]. Recently it has been proposed that, in PD as well as in AD and multiple sclerosis, the complex and synergistic interaction between neuroinflammatory processes and mitochondria may result in the generation of a self-renewing vicious cycle, ultimately leading to neuronal death, with the mitochondria as a crucial link between neuroinflammation and neurodegeneration [146]. Mitochondrial dysfunction has been demonstrated to be an early pathological event in PD and other neurodegenerative disease, but also in brain aging. Interestingly, in animal models for these conditions, it has been shown an improvement of mitochondrial functions after administration of polyunsaturated fatty acids (PUFAs) and in particular the omega-3 docosahexaenoic acid (DHA; [147]). The brain, after adipose tissue, is the organ richest in lipids, and PUFAs are essential components of neuronal and glial cell membranes. PUFAs intake regulates the production of pro- and anti-inflammatory cytokines, with omega-3 FAs having anti-inflammatory properties and omega-6 FAs having pro-inflammatory effects. Therefore, depending on which PUFAs are present in the diet, neuroinflammation is lowered or enhanced. This could explain the protective role of omega-3 in mitochondrial functions, aging and neurodegenerative diseases [148]. Accordingly, in animal models, it has been demonstrated that DHA from dietary sources is rapidly incorporated into mitochondrial membranes and that it is essential for mitochondrial functions [149]. In general, the relationship between PD and diet is controversial and the discrepancies can be attributed to different methodological approaches, genetic and gender-specific factors. However, recent studies indicate a potential effect of PUFA in reducing the risk of PD while high cholesterol and arachidonic acid intakes may elevate this risk [150,151]. Further studies are necessary to verify how the intake of dietary fat affects PD risk.

#### 5.2.3. Bipolar Disorders and Schizophrenia

It is important to underline that a disturbance of energy metabolism has been found frequently associated with neuropsychiatric diseases as bipolar disorders and schizophrenia. In particular, different kinds of mitochondrial dysfunction are linked to these illnesses, including decreased mitochondrial respiration, changes in mitochondrial morphology and increased levels of mitochondrial DNA mutations [152]. Accordingly, mitochondrial disorders are often associated with cognitive decline and psychotic and affective symptoms [153]. Indeed, preclinical and clinical studies targeting mitochondria have been performed to treat bipolar disorders. These mitochondrial agents seem to have beneficial effects and are mostly well tolerated [154]. Thus, investigating the mechanisms underlying neuroinflammation and mitochondrial functions may represent a crucial step in developing an innovative therapeutic approach for neuropsychiatric diseases.

### 5.3. Overnutrition and Serotonin Signaling

A role of serotonin (5-HT) in the regulation of energy balance is well established not only in the central nervous system but also in peripheral tissue [155,156]. It is noteworthy that the action of 5-HT affecting feeding behaviour and obesity in the CNS seems to be independent from 5-HT mechanisms acting in periphery since 5-HT cannot pass the blood–brain barrier [157,158,159,160,161,162]. The brain 5-HT acts on the hypothalamus, a pivotal CNS area for the integration of energy balance signals, and activates pro-opiomelanocortin (POMC)-expressing neurons through 5-HT receptor 2C (5-HT2CR), while it inhibits neuropeptide Y/agouti-related peptide (NPY/AgRP)-expressing neurons via 5-HT1BR to induce satiety and to enhance energy expenditure [156].

5-HT is known to exert morphogenic actions on the brain, influencing several neuronal processes such as neurogenesis, cell migration, axon guidance, dendritogenesis and synaptogenesis. In particular, the activation of 5-HT7R modulates synaptic plasticity, contributing to the establishment of brain connectivity during embryonic and early postnatal life [163,164]. Thus, 5-HT could be considered another important factor linking energy homeostasis and brain plasticity.

HFD treatment exerts regionally specific effects on brain 5-HT levels. In the hypothalamus of HFD-fed rats, 5-HT levels selectively decrease, and this effect is probably linked to the higher circulating leptin observed in these animals [165]. Accordingly, other studies provided evidence that leptin regulates the local release of 5-HT in hypothalamus [166,167]. The HFD effects on brain 5-HT levels is region-specific. Indeed, in the hippocampus of mice fed with HFD, decreased levels of 5-HT were observed, accompanied with anxiogenic, depressive-like symptoms [168]. Interestingly, in the same brain region of a mice model of diet-induced obesity, decreased expression levels of 5-HTRs were observed, particularly marked for 5-HT1AR [169,170,171]. It is worth noticing that the effects of HFD on 5-HT metabolism in the hippocampus are controversial, since other authors showed an increase in 5-HT levels in this region of HFD-fed rodents and suggested that this increase could be due to the greater excitability of serotonergic neurons present in the hippocampus caused by decrease in the levels of GABA [165]. Indeed, decreased GABA levels were reported to occur in the hippocampus of rats treated with HFD [78].

Interestingly, leptin and adiponectin may also play a key role in the cross talk between metabolic status and neurological disorders, since both adipokines modulate a range of neuropathological events, including amyloidogenesis, tau hyperphosphorylation, neuroinflammation, oxidative stress, synaptic dysfunction and cognitive impairment [172,173].

Only approximately 5% of the total body content of 5-HT is located in the CNS, while the remaining part is synthesized and stored in peripheral tissues, particularly in the gastrointestinal epithelium, where it is mainly produced by enterochromaffin cells of the gut mucosa [163].

Peripheral 5-HT plays an important role in glucose and lipid metabolism [174,175,176,177,178,179] and may act as mediator of neuro-behavioural dysfunction induced by metabolic disorders [180]. Increased peripheral 5-HT is associated with obesity [181], while inhibition of peripheral 5-HT synthesis reduces metabolic dysfunction related to obesity by promoting thermogenic activity in brown adipose tissue via activation of uncoupling protein 1 (UCP1)-mediated thermogenesis [182,183]. Conversely, it was observed that intraperitoneal injection of 5-HT in mice prevents obesity by inducing an increase of mitochondria activity and energy metabolism in skeletal muscle through activation of 5-HT2AR and 5-HT7R and elevation of peroxisome proliferator-activated receptor gamma coactivator 1-alpha (PGC) mRNA expression [176].

Interestingly, the selective inhibition of gut-derived 5-HT can significantly reverse the decrease of 5-HT1AR expression levels observed in the hippocampus of mice fed with HFD, suggesting that inhibiting intestinal 5-HT could ameliorate neurological disorders elicited by the metabolic dysfunction [180]. These results are difficult to explain considering that peripheral 5-HT cannot cross the blood–brain barrier. Thus, it is possible to hypothesize that peripheral 5-HT indirectly affects brain functions, for instance, through its modulatory effects on leptin and ghrelin and through diverse inflammatory cytokines which are able to cross the blood–brain barrier, mediating satiety and hunger signals [156,184,185].

## 6. Conclusions

Remarkable progress has been made in the past two decades regarding the effects of overnutrition-related chronic low-grade inflammation on the onset of obesity comorbidities. Interestingly, recent data indicate that this systemic inflammation affects also the CNS, leading to neurological diseases. In our review, we focused on the concept that overnutrition acts on the CNS, anticipating the aging effects and leading to early onset of neuroinflammation particularly pronounced in the synaptic regions. Intriguingly, neuroinflammation and oxidative stress are tightly linked to dysfunction of synaptic mitochondria that do not satisfy the high energy demands required at synapses, leading to altered synaptic plasticity and neurodegeneration. From this point of view, synaptic mitochondria can be considered as a potential target for treating metabolic dysfunctions and neurological disorders.

## Abbreviations

HFD	High fat diet
ROS	Reactive oxygen species
AMP	Adenosine monophosphate
BDNF	Brain derived neurotrophic factor
ER	Endoplasmic reticulum
IR	Insulin resistance
FFA	Free fatty acids
TLR4	Toll-like receptor 4
NF-κB	Nuclear factor kappa-light-chain-enhancer of activated B cells
TNFα	Tumour necrosis factor-α
IL-6	Interleukin-6
AMPK	Adenosine monophosphate activated protein kinase
ACC	Acetyl-CoA carboxylase
CPT	Carnitine palmitoyl-transferase
CNS	Central nervous system
IKK	IkB kinase
ncRNA	Non-coding RNA
POMC	Pro-opiomelanocortin
PFC	Prefrontal cortex
GABA	γ-aminobutyric acid
miRNAs	microRNAs
LTP	Long-term potentiation
IEGs	Immediate early genes
AD	Alzheimer’s disease
APP	Amyloid precursor protein
mTOR	Mammalian target of rapamycin
5-HT	Serotonin
UCP1	Uncoupling protein 1
PGC	Peroxisome proliferator-activated receptor gamma coactivator 1-alpha
PINK1	PTEN-induced putative kinase 1
DHA	Docosahexaenoic acid
PUFAs	polyunsaturated fatty acids
PD	Parkinson’s disease
NPY/AgRP	neuropeptide Y/agouti-related peptide
